# Structural Effects on Compressive Strength Enhancement of Cellular Concrete During the Split Hopkinson Pressure Bar Test

**DOI:** 10.3390/ma18030552

**Published:** 2025-01-25

**Authors:** Ling Zhou, Zhiping Deng, Junru Ren, Yuhao Zhu

**Affiliations:** Army Logistics Academy, 20 North First Road, College Town, Shapingba District, Chongqing 401311, China

**Keywords:** impact, cellular concrete, mechanical properties, SHPB test, numerical modeling, structural effect, lateral inertia confinement effect, interface friction effect

## Abstract

In recent years, a kind of novel cellular concrete, fabricated by spherical saturated superabsorbent polymers, was developed. Its compressive behavior under high strain rate loadings has been studied by split Hopkinson pressure bar equipment in previous research, which revealed an obvious strain rate effect. It has been found by many researchers that the dynamic increase factor (DIF) of compressive strength for concrete-like materials measured by SHPB includes considerable structural effects, which cannot be considered as a genuine strain rate effect. Based on the extended Drucker–Prager model in Abaqus, this paper uses numerical SHPB tests to investigate structural effects in dynamic compression for this novel cellular concrete. It is found that the increment in compressive strength caused by lateral inertia confinement decreases from 5.9 MPa for a specimen with a porosity of 10% to 2 MPa for a specimen with a porosity of 40% at a strain rate level of 70/s, while the same decreasing trend was found at other strain rate levels of 100/s and 140/s. The lateral inertia confinement effect inside the cellular concrete specimen can be divided into the elastic development stage and plastic development stage, bounded by the moment dynamic stress equilibrium is achieved. The results obtained in this research can help to obtain a better understanding of the enhancement mechanism of the compressive strength of cellular concrete.

## 1. Introduction

**Figure 1:**
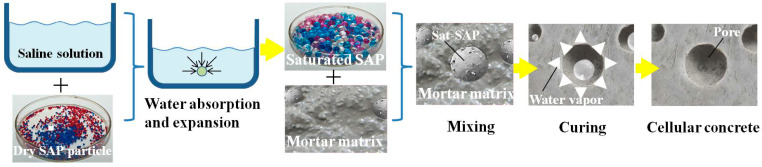
The schematic formation process of cellular concrete (the black arrow represents the flow direction of water, and the white arrow represents the flow direction of water vapor) [3].

In recent years, a new kind of cellular concrete with round pores, 4–8 mm in diameter [1], has been developed. The formation process (as shown in Figure 1) of this novel cellular concrete was elucidated in [2,3]. This cellular concrete has a heat insulation reduction of 0.316 W/(m·K), a noise reduction coefficient reaching 0.348, and, at the same time, an internal curing effect [4,5,6,7]. Moreover, as a kind of lightweight concrete [8,9,10,11,12,13], like foam concrete, it can be used as an energy buffering material in airport stopping pads and so on [14,15,16,17,18,19]. Based on this kind of novel cellular concrete, the shock tube test was conducted by Ren et al. [20] and indicated that BSs (BFRP-reinforced cellular concrete slabs) demonstrated better wave-attenuating abilities than SPs (steel-reinforced normal concrete slabs).

To facilitate its application in structure protection on remote islands, the dynamic mechanical responses on impact or blast loading of this cellular concrete need to be investigated, while the current relevant research is lacking. The previous research [2] on the compressive behavior under shock loadings by split Hopkinson pressure bar (SHPB) equipment of this cellular concrete revealed a clear strain rate dependence, and the dynamic increase factor of compressive strength increases when the porosity is promoted. Namely, the dynamic increase factor of the compressive strength (DIF-fc) for specimens with high macroporosity is about 15.6%~31.8% higher than that for specimens with relatively low macroporosity at selected high strain rates. Other experimental studies [21,22] on similar cellular concrete revealed a consistent pattern to that described in reference [2].

He and Gao et al. [21] conducted impact tests on foam concrete using an SHPB device. The results indicated that the compressive strength has a significant correlation with the strain rate. The DIF of the compressive strength decreased with increasing density for two types of foam concrete, i.e., with an increase in the macroporosity, the DIF of the compressive strength increased from about 2.1 to 2.8 for specimens with fly ash. Feng and Zhou et al. [22] tested foam concrete using an SHPB device, which also revealed an obvious strain rate effect for compressive strength; moreover, this strain rate effect is density-dependent. For example, when the loaded specimen has a strain rate of 60/s, the increment ratio of the strength gradually increases from 1.3 to 2.23 with density ascending from 300 kg/m^3^ to 700 kg/m^3^.

For concrete materials, the steep increase in compressive strength in the SHPB test, in contrast to the static loading test, may be ascribed to the structural effect (mainly including lateral inertia, interface friction, etc.) [23,24,25,26,27,28,29,30,31,32,33,34,35,36,37,38,39,40]. In relation to numerical simulation, Li et al. [25] simulated the SHPB test, showing that the strength enhancement is strongly influenced by the lateral inertia confinement during the SHPB test. Ma et al. [28] adopted the smoothed particle hydrodynamics (SPH) method and pressure-dependent J-H constitutive model to study the strain rate effect for heterogeneous brittle materials. The results pointed out that the structural effects, such as inertial confinement, contribute to the explanation of the strain rate effect. Zhou et al. [29] obtained numerical results to confirm the inertial confinement effect in the compressive strength enhancement of the specimen. Using FEA (Finite Element Analysis), Kim et al. [30] confirmed that the radial inertia confining effect causes an increase in the compressive strength for the SHPB tests. In experimental research, Zhang et al. [33] conducted SHPB tests, and the obtained results indicated a close relationship between the dynamic increase factor of compressive strength and radial inertia. As for the analytical analysis of a metal specimen, the inertia effects in an SHPB test were analytically derived by Davies and Hunter [35] and Gorham [36]. As for brittle specimens, Forrestal et al. [37] derived a formula, indicating the inertia effect is one of the factors that induced the DIF increment of concrete.

Crack propagation theory has been introduced by [2] to elucidate the experimental results showing that the dynamic increase factor increases with porosity; however, the structural effects and mechanism have not been investigated. Further research was conducted in this paper to reveal contributions to the structural effects of cellular concrete in SHPB tests, since a misinterpretation of structural effects may lead to non-conservative design of structures.

In this paper, finite element (FE) simulations using the extended Drucker–Prager model (D-P model) in Abaqus are performed to shed light on the structural effects (mainly the lateral inertia confinement effect and interface friction effect) on compressive strength enhancement of the cellular concrete in the SHPB test and their mechanisms.

## 2. Methodology

**Figure 2:**
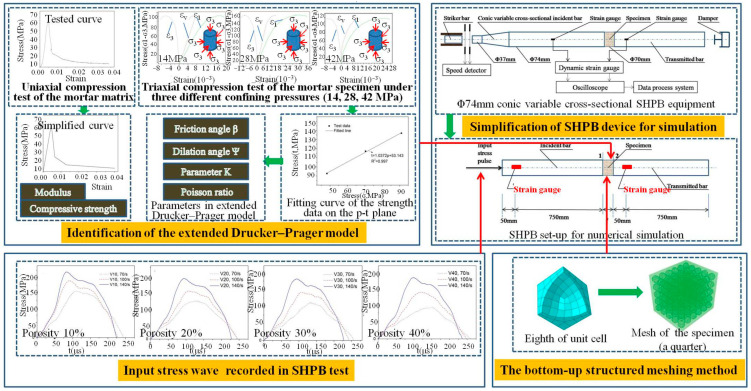
Schematic diagram of the numerical simulation process (*ε*_1_ is axial strain along length direction of the cylinder specimen, *ε*_3_ is lateral strain along the radial direction of the cylinder specimen and *ε_v_* is the volumetric strain of the cylinder specimen; *σ*_3_ is the confining pressure in triaxial compression test).

The novel cellular concrete is mainly composed of two parts: mortar matrix and spherical pores. Like concrete, the mortar matrix has a hydrostatic pressure correlation in its strength. The hydrostatic pressure correlation is the internal reason leading to the lateral inertial confinement effect of concrete-like materials in the SHPB test [25]. In order to investigate the non-strain-rate effects on the compressive strength of this novel cellular concrete with millimeter-size isolated spherical pores, the same numerical approach as in references [25,26] was taken, and it was composed of four parts (as shown in Figure 2).

1. Simplification of SHPB device for simulation

The SHPB equipment utilized in [2] is simplified, in this numerical simulation, as follows: a conic variable cross-sectional pressure bar is simplified as a prismatic bar; the lengths of the incident and transmitted bars are both shortened from 3200 mm to 800 mm; the circular sections of the pressure bars and specimens are simplified as square sections. The location of the strain gauge is displayed in Figure 2, by which the stress wave is obtained to reconstruct stress–strain curves of the specimens through the “two-wave method” (as shown in Equation set (1)) [2].

2. Identification of the extended Drucker–Prager model

In numerical simulations, the strain rate effect of the mortar matrix material itself is not considered. The Drucker–Prager model of the mortar matrix can reflect the hydrostatic pressure correlation of the strength, meanwhile the strain rate effect can be set separately [25], that is, the influence of structural effects (such as the lateral inertia confinement effect) on the dynamic mechanical behavior for specimens with different porosities can be separately simulated. To identify the extended Drucker–Prager model, the friction angle β, dilation Ψ, parameter K, and Poisson ratio are obtained through triaxial compression test [25] of the mortar specimen with the same composition and mix proportion as the mortar matrix of the cellular concrete specimens in reference [2]. The modulus, compressive strength, and uniaxial compressive stress–strain curve for extended Drucker–Prager model are obtained through uniaxial compression tests [25] of the same mortar specimen as in the triaxial compression test.

3. Input stress wave recorded in SHPB test

The incident stress waves obtained in the SHPB test in reference [2], which are recorded for the cellular concrete specimens with four different porosities (10%, 20%, 30%, and 40%, respectively) at three different strain rate levels (70/s, 100/s, and 140/s respectively), are directly used as the input wave of the numerical simulation to act on the loading end of the incident bar.

4. The bottom-up structured meshing method

The meso-element equivalent method based on the random specimen model in reference [3] is not adopted; instead, the spherical pores are evenly distributed in the specimens in this paper. The trial calculations revealed that the meshing quality of the matrix around pores is the principal factor that affects the accuracy and effectiveness of dynamic calculation results. The meshing quality must be strictly guaranteed to ensure that the calculation goes on and no ‘hourglass’ comes up. Thus, the bottom-up structured meshing method is employed in this paper, and the C3D8R element is taken to mesh the incident bar, transmitted bar, and specimens. The mesh density is determined by the method of multiplicity. Parameters of the finite element model for the cellular concrete specimens are displayed in Table 1.

## 3. Results and Discussion

### 3.1. Dynamic Stress Equilibrium

**Figure 3:**
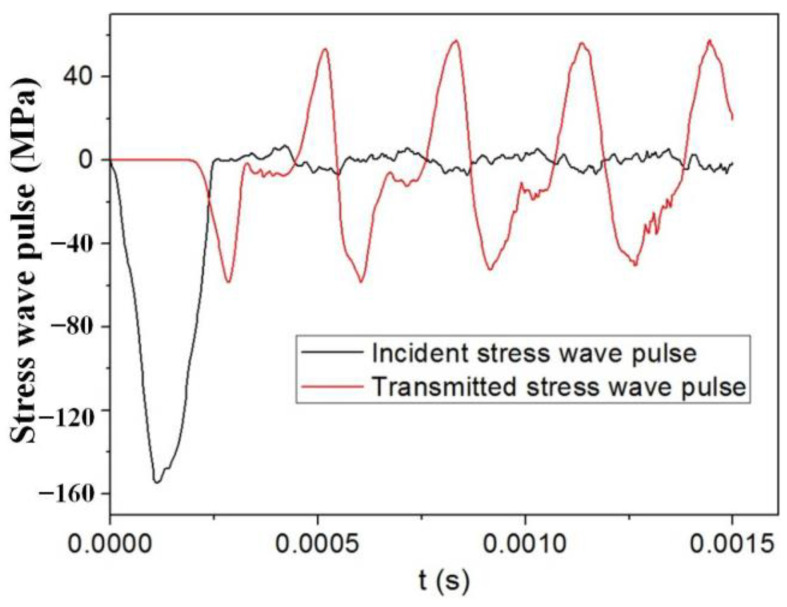
Typical pulse signal of the incident and transmitted stress wave pulse from numeric model.

After the intercepted transmitted stress wave is aligned with the incident stress wave in Figure 3, the stress–strain curve of the specimen can be reconstituted by using the “two-wave method” [2]:

(1) $$\left\{\begin{array}{c} \sigma_{c}(t)=\frac{E_{b}A_{b}}{A_{c}}\varepsilon_{t}(t)\\ \varepsilon_{c}(t)=\frac{2C_{b}}{l_{c}}\int_{0}^{t}[\varepsilon_{i}(t)-\varepsilon_{t}(t)]dt\\ {\dot{\varepsilon }}_{c}(t)=\frac{2C_{b}}{l_{c}}[\varepsilon_{i}(t)-\varepsilon_{t}(t)] \end{array}\right.$$

In Equation set (1), $\sigma_{c}(t)$/Pa, $\varepsilon_{c}(t)$/1, and ${\dot{\varepsilon }}_{c}(t)$/s^−1^ are the time-dependent nominal stress, nominal strain, and nominal strain rate of the concrete specimens during deformation, respectively; $A_{b}$/m^2^, $C_{b}$/(m/s), and $E_{b}$/Pa are the cross-sectional area, longitudinal stress wave velocity, and elastic modulus of the alloy steel bars, respectively; $A_{c}$/m^2^ and $l_{c}$/m are cross-sectional area and length of the concrete specimen, respectively.

The “two-wave method” is based on the assumption that the specimen is in dynamic stress equilibrium during the SHPB test. The equilibrium can be examined by comparing the stresses *σ*_1_(t) (the stress at the end face of specimen between specimen and incident bar) and *σ*_2_(t) (the stress at the end face of specimen between specimen and transmitted bar). If stresses *σ*_1_(t) and *σ*_2_(t) are in reasonable agreement, only then is the specimen in dynamic stress equilibrium and the use of Equation set (1) valid.

**Figure 4:**
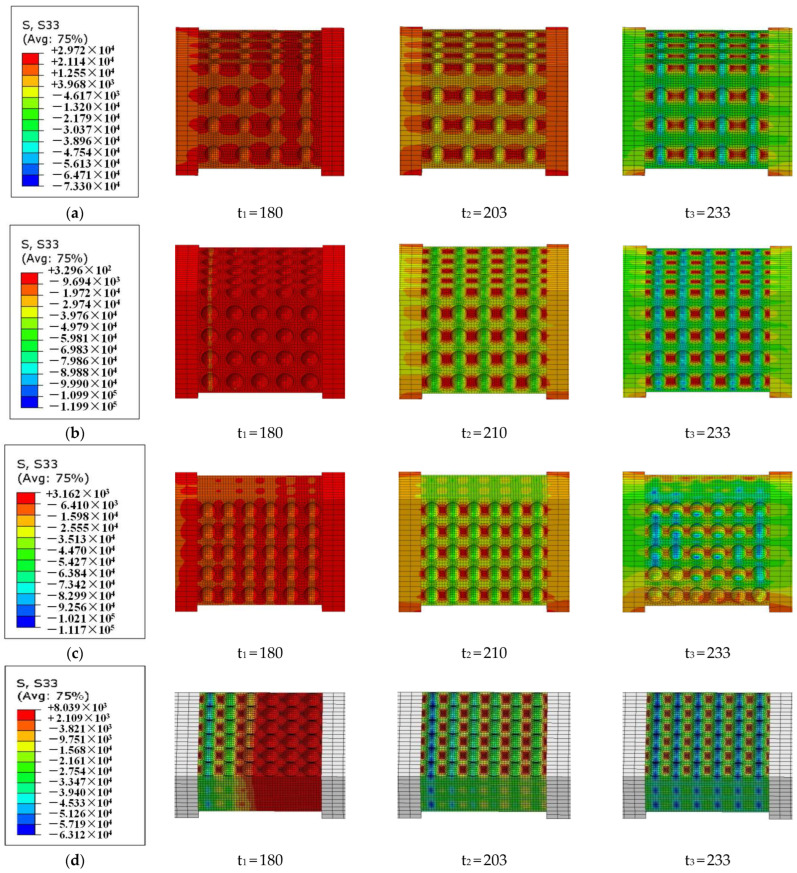
Stress nephogram of S33 (along the axial direction of the pressure bar) for specimens with different porosities ((**a**) 10%, (**b**) 20%, (**c**) 30%, and (**d**) 40%) at different moments (μs).

The stress nephogram of the stress component S33 (the stress component in the specimen along the axial direction of the pressure bar) at different times can be used to analyze the process from the beginning of loading to the dynamic stress equilibrium of the specimen. As shown in Figure 4, the incident stress wave begins to load the specimen at moment t_1_, time t_3_ is the moment when the specimen initially achieves the dynamic stress equilibrium, and time t_2_ is the moment between time t_1_ and t_3_. And for the specimens with different porosities, it takes about 53 μs to achieve the dynamic stress equilibrium after the specimen is loaded by the incident wave, which is basically consistent with the conclusion (50 μs) obtained by analyzing the strain gauge signal of the SHPB test in [2].

It is worth noting that, in Equation set (1), *A_c_* is the cross-sectional area of the concrete specimen; in fact, the efficient area *A_e_* is smaller than *A_c_* for the cellular concrete since spherical pores are distributed in the mortar matrix. The stress areas in Figure 4 consist of compressive stress regions and tensile stress regions. The compressive stress calculated from Equation set (1) is actually less than the stress in the compressive stress region, which is a nominal stress. Thus, the dynamic stress equilibrium in the SHPB test for a cellular concrete specimen is a nominal concept considering the specimen as a whole part.

### 3.2. Lateral Confinement Effect

#### 3.2.1. Effect of the Lateral Inertia Confinement

**Figure 5:**
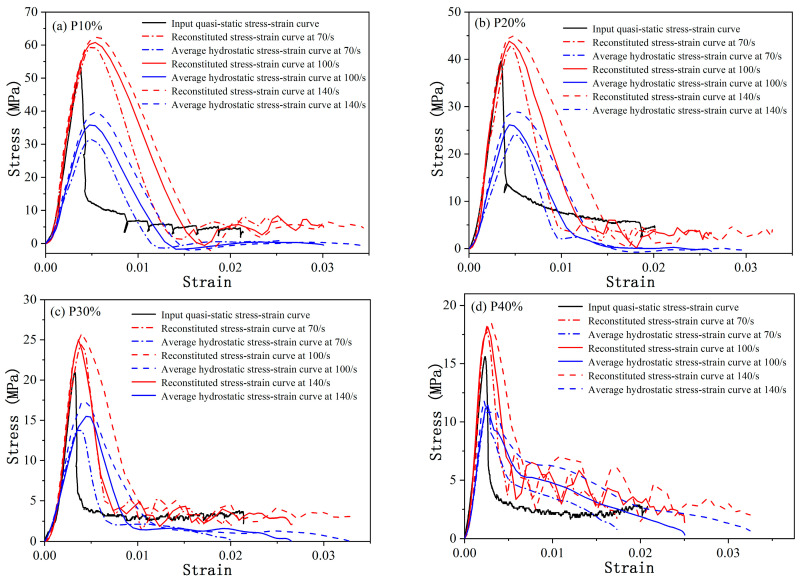
Reconstituted stress–strain and average hydrostatic pressure curves of the cellular concrete with porosity *p* at strain rate levels of 70 s^−1^, 100 s^−1^, and 140 s^−1^ (**a**) for a porosity of 10%, (**b**) for a porosity of 20%, (**c**) for a porosity of 30%, and (**d**) for a porosity of 40%.

The reconstituted stress–strain curves and corresponding average hydrostatic pressure obtained through simulation, as shown in Figure 5, only consider the inertia effect (mainly lateral inertia confinement effect), ignoring the interface friction effect. The rising stage of the reconstituted stress–strain curves overlaps well with that of quasi-static curves before peak stress at different strain rates. As for the mean hydrostatic pressure curves in Figure 5, the point of peak stress is consistent with that of corresponding reconstituted curve, indicating a close relationship between hydrostatic pressure and compressive strength at a high strain rate. Thus, the enhancement of compressive strength for the reconstituted curve at a high strain rate may contribute to the lateral inertia confinement effect in the SHPB test.

**Figure 6:**
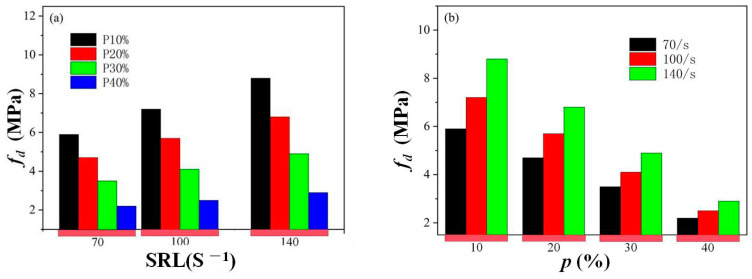
Variation in increment in compressive strength *f*_cl_ with strain rate level (**a**) and porosity (**b**).

**Figure 7:**
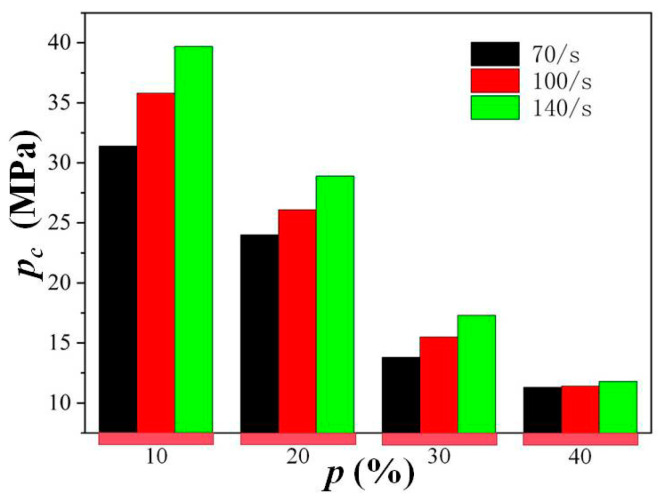
Variation in the peak value of average hydrostatic pressure *p_c_* with porosity *p* at strain rate levels of 70 s^−1^, 100 s^−1^, and 140 s^−1^.

Figure 6 manifests a clear trend that *f_cl_* (the increment between quasi-static and reconstituted compressive strength) increases with the increase in the strain rate and decreases with the rise in porosity *p*. At a strain rate level (SRL) of 70/s, the increment in compressive strength caused by lateral inertia confinement *f_cl_* drops from 5.9 MPa for a specimen with a porosity of 10% to 2 MPa for a specimen with a porosity of 40%. At a strain rate level of 100/s, the increment in compressive strength caused by lateral inertia confinement *f_cl_* drops from 7.1 MPa for a specimen with a porosity of 10% to 2.4 MPa for a specimen with a porosity of 40%. At a strain rate level of 140/s, the increment in compressive strength caused by lateral inertia confinement *f_cl_* drops from 8.8 MPa for a specimen with a porosity of 10% to 3 MPa for a specimen with a porosity of 40%. Thus, the lateral inertia confinement effect increases with strain rate; however, high porosity can weaken this inertia effect. Figure 7 also verifies this phenomenon from another perspective, in which the *p_c_* values are almost the same for specimens with a porosity of 40% at a strain rate level from 70/s to 140/s, indicating a relatively small hydrostatic pressure and corresponding strength enhancement provoked by a higher strain rate for cellular concrete with high porosity. This phenomenon is consistent with the speculation in [2] that the inertia effect would be relatively less significant for cellular concrete since the confining pressure applied on the central core concrete may be smaller, contributing to the mass loss of the surrounding concrete induced by pores in the concrete. Thus, the mechanism of the crack-path-altering effect can be further confirmed to explain the higher dynamic increase factor for specimens with high porosity than for specimens with relatively low porosity at the selected high strain rate, which is found in the SHPB test for this kind of cellular concrete [2].

#### 3.2.2. Mechanism of Lateral Inertial Confinement Effect

1. Stress and strain analysis

**Figure 8:**
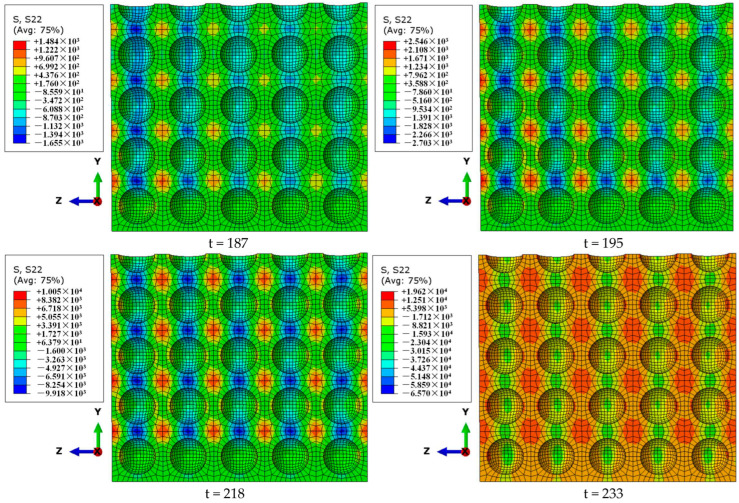

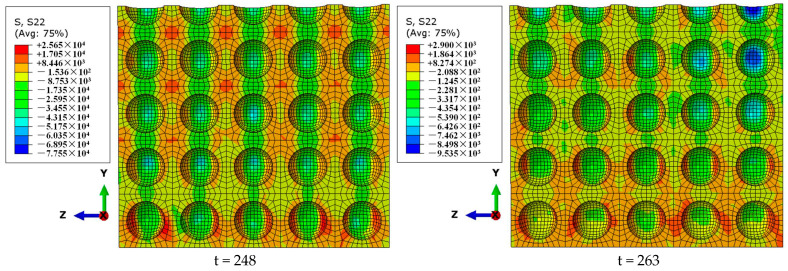
Stress nephogram of S22 on the cross-section X = b/2 (b is 1/2 the width of the specimen along the axial direction of the pressure bar) at different times (μs) for a specimen with a porosity of 20%.

As shown in Figure 8, the time t = 187 μs is the moment that the stress wave begins to spread across the specimen. The stress nephogram of S22 (along the *Y*-axis, namely the radial stress nephogram along the *Y*-axis direction on the surface) at this moment can be roughly divided into three regions: the blue radial compressive stress region, the orange radial tensile stress region, and the green transition region. From the moment t = 187 μs to the moment t = 233 μs (the moment dynamic stress equilibrium is reached), the local radial compressive stress region and radial tensile stress region gradually expand from the incident end to the transmitted end. At the moment t = 233 μs of dynamic stress equilibrium, the local radial compressive stress region, the local radial tensile stress region, and the local transition region are uniformly distributed inside the specimen. At this time, the size and strength of each local stress region are basically the same, and there is no nonlinear distribution from the inside of the specimen (the upper-side line of each section in Figure 8) to the edge (bottom of each section in Figure 8). Therefore, this stage can be classified as the elastic development stage of radial stress. From the moment of dynamic stress equilibrium (t = 233 μs) to the moment of peak stress (t = 263 μs), the size and stress value of each region develop from a uniform distribution to a non-uniform distribution. The size and strength of the radial compressive stress region gradually shift to the center of the specimen, and the radial tensile stress region gradually shifts to the edge of the specimen. When t = 263 μs, the peak stress is reached, and the size and strength of the local radial compressive stress region show a decreasing distribution from the inside of the specimen to the edge. In this case, the local radial tensile stress region is mainly distributed in the region with a certain thickness on the edge of the specimen. This stage can be classified as the plastic development stage of radial stress.

Therefore, during the period from the moment the stress wave enters the specimen to the time peak stress is reached, the lateral inertia confinement inside the specimen can be roughly divided into the elastic development stage and the plastic development stage, which are bounded by the moment when the specimen reaches the dynamic stress equilibrium. In the elastic development stage, the lateral inertia confinement develops to the uniform distribution of each local region. In the plastic development stage, the local regions of the lateral inertia confinement develop a decreasing distribution from the inside of the specimen to the edge.

**Figure 9:**
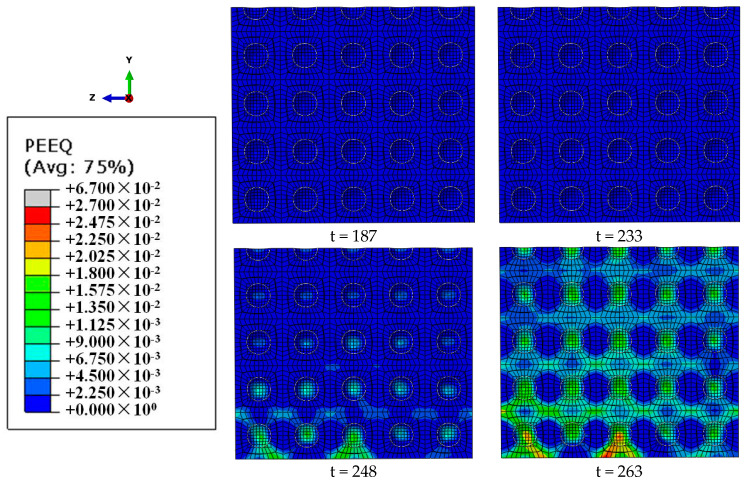
Distribution graph of equivalent plastic strain PEEQ on the cross-section X = b/2 (b is 1/2 the width along the axial direction of the pressure bar) at different moments (μs) for a specimen with a porosity of 20% at a 70/s strain rate.

The development of plastic strain in the two stages can be directly reflected from the time t = 187 μs of the stress wave entering the specimen to the time t = 233 μs of dynamic stress equilibrium (the first stage) and from the time t = 233 μs of dynamic stress equilibrium to the time t = 263 μs of peak stress being achieved (the second stage). In ABAQUS, PEEQ (PE represents plastic strain component and EQ represents equivalent quantity in ABAQUS) is the parameter used to describe the accumulation of strain in a material during plastic deformation. When the equivalent plastic strain PEEQ is greater than 0, it indicates that the material has yielded and entered the plastic deformation stage. In the first stage (Figure 9), the PEEQ of each part of the specimen is basically zero, and the specimen is basically in the elastic stage. In the second stage, after the dynamic stress equilibrium is achieved, the equivalent plastic strain PEEQ begins to originate and develop. The plastic strain is mainly generated at the edge and then propagates to the inside of the specimen. It is due to the radial inertia generated by the plastic expansion of the edge plastic zone and then propagates to the inside and the edge of the specimen; the size and strength of the local radial compressive stress region show a decreasing distribution from the inside of the specimen to the edge at the moment t = 263 μs that peak value of stress is reached.

Figure 9 directly confirms the “two-stage” development mechanism, and the effect of lateral inertia confinement can be divided into two stages: the elastic development stage before the dynamic stress equilibrium is achieved and the plastic development stage after the dynamic stress equilibrium moment. In the elastic development stage, the lateral inertia confinement develops with a uniform distribution in each local area, and the lateral inertia confinement is small. In the plastic development stage, the local regions of the lateral inertia confinement develop a decreasing distribution from the inside of the specimen to the edge, and the lateral inertia confinement is larger. Since the plastic development stage is the stage where the peak stress is reached and the strain rate level is determined [2], the increase in compressive strength caused by the lateral inertia confinement effect is mainly attributable to the propagation of the lateral confinement to the interior of the specimen, which starts from the plastic expansion of the plastic zone at the edge of the specimen.

**Figure 10:**
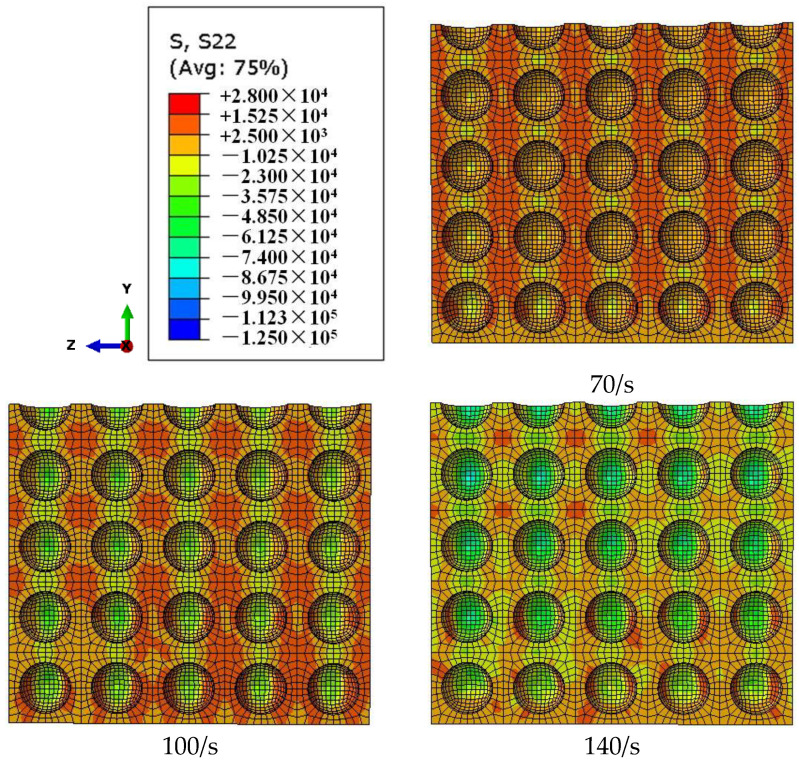
Stress distribution graph of S22 on the cross-section X = b/2 under different strain rates (70/s, 100/s, and 140/s) for a specimen with a porosity of 20% at time t = 233 μs.

**Figure 11:**
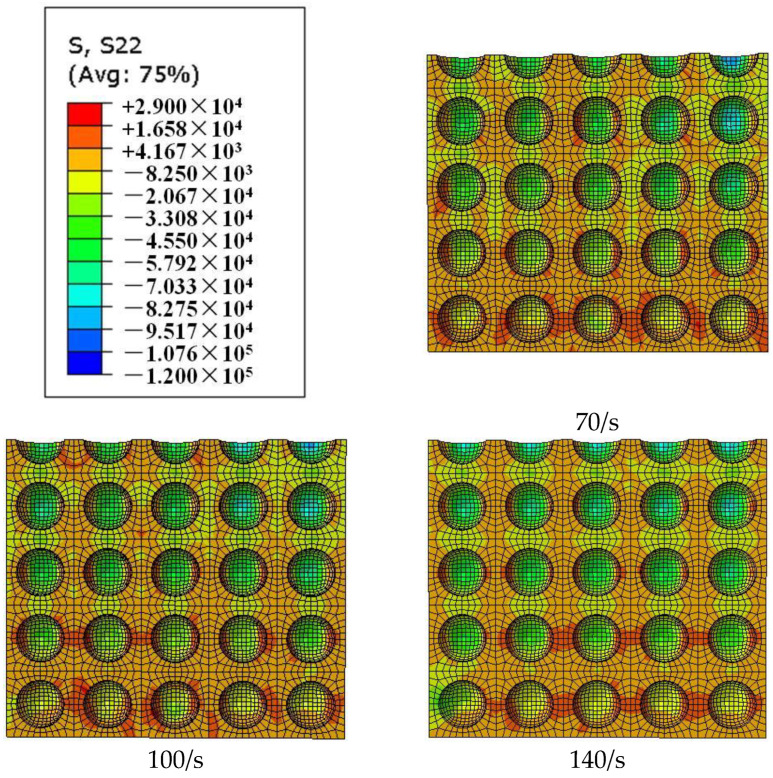
Stress distribution graph of S22 on the cross-section X = b/2 under different strain rates (70/s, 100/s, and 140/s) for a specimen with a porosity of 20% at the time (t = 248 μs) peak stress is reached.

Figure 10 demonstrates that, in the elastic development stage of the lateral inertia confinement, with the increase in the strain rate, the radial compressive stress region and stress value gradually increase, while the radial tensile stress region and its value gradually decrease. As can be seen from Figure 11, the radial compressive stress region transfers to the interior of the specimen in the plastic development stage with the increase in the strain rate, while the radial tensile stress region transfers to the edge of the specimen. The higher the degree of the transfer developed, the stronger the lateral inertia confinement effect caused by plastic expansion. Figure 10 and Figure 11 reveal the internal mechanism of the lateral inertial confinement effect increasing with the increase in the strain rate when the porosity is the same.

**Figure 12:**
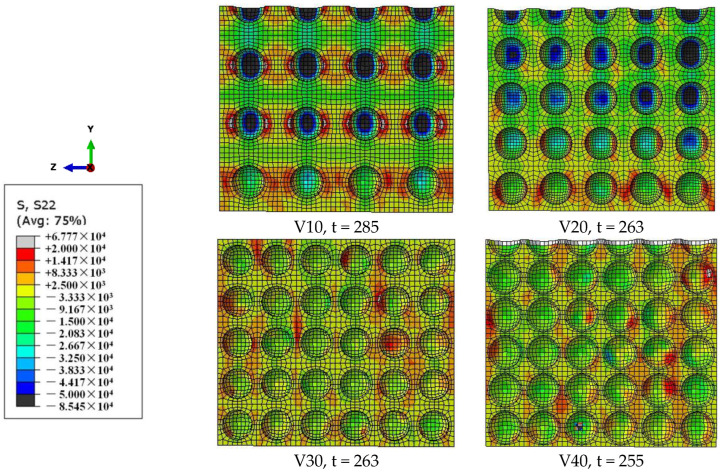
Stress distribution graph of S22 on the cross-section X = b/2 of specimens with different porosities (10%, 20%, 30%, and 40%) under a strain rate of 70/s at the time (μs) peak stress is reached.

As can be seen from Figure 12, at the same strain rate level, the radial compressive stress region has a more significant decreasing distribution trend from the inside of the specimen to the edge with a decrease in porosity. Meanwhile, the compressive stress value in the radial compressive stress region increases significantly with a decrease in porosity. Figure 12 reveals the internal mechanism of the lateral inertial confinement effect decreasing with the increase in porosity at the same strain rate level.

2. Mechanism of lateral inertial confinement effect

**Figure 13:**
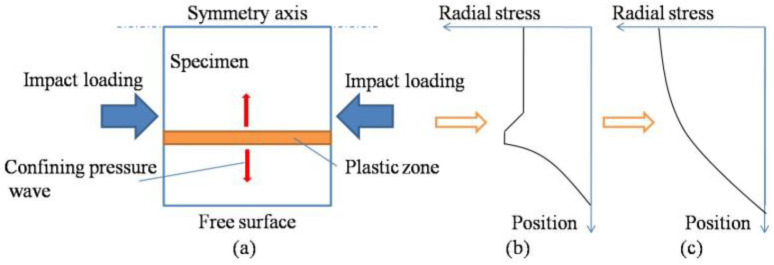
Schematic diagram of lateral inertia confinement.

As can be concluded from the stress and strain analysis, in an SHPB test of cellular concrete materials, the lateral inertia confinement caused by axial inertia cannot be explained by the elastic theory, but the mechanism should be analyzed by the plastic-flow-related theory. When concrete materials enter the plastic stage, the strain increases rapidly with the increase in load, and the axial strain will cause the rapid growth of transverse strain. The rapid growth of strain results in a larger radial inertia confining pressure since the derivative of strain rate with respect to time is the amount related to inertia. The plastic expansion of the plastic zone inside the specimen generates transverse inertia, and the radial confining pressure will simultaneously propagate to the inside and edge of the specimen, as shown in Figure 13.

Figure 13a shows a specimen subjected to impact loading in the horizontal direction, in which the upper end of the vertical direction is the symmetry axis of the specimen (the central end in the radial direction), the lower end is the free surface at the edge of the specimen, and the orange band is an area where plastic deformation occurs. After the local area enters the plastic deformation phase (as shown by the orange band), the plastic zone becomes the disturbance source of the confining pressure wave inside the specimen, and the radial confining pressure generated there propagates to the central end and the free edge simultaneously (as shown by the red arrow in Figure 13a). The stress of the confining wave is doubled after reflection at the central end and unloaded to zero after reflection at the free end. Thus, the distribution curve of radial confining pressure with the radial position at a certain moment as shown in Figure 13b is formed. When most or even all regions enter the stage of plasticity, the radial confining pressures generated by disturbance sources in each plastic zone are superimposed on each other, and finally, the radial confining pressure as shown in Figure 13c is distributed in a parabolic form along with the radial position. That is, the lateral confinements produced by the inertia effect in the specimen show a parabolic decreasing trend from the center to the edge.

### 3.3. Effects of Interface Friction

**Figure 14:**
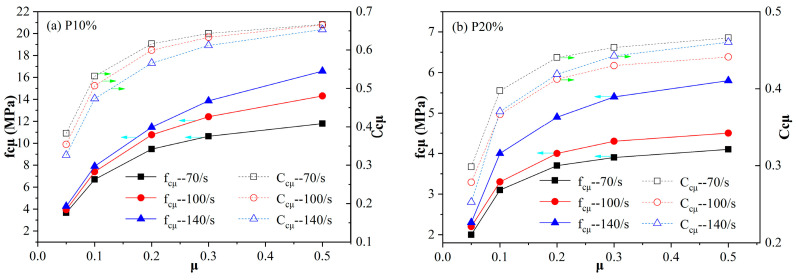

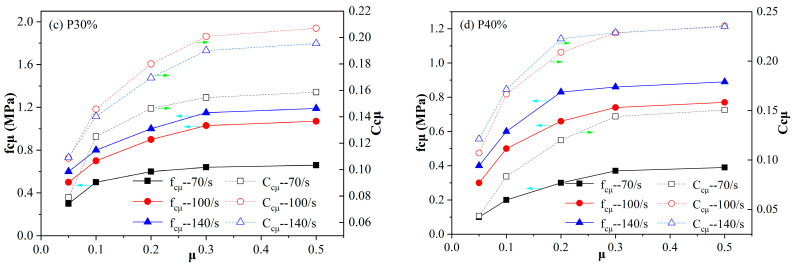
Variations in *f_cμ_* (the label blue arrows point to) and *C_cμ_* (the label green arrows point to) with interface friction coefficient *μ* for specimens with different porosities ((**a**) 10%, (**b**) 20%, (**c**) 30%, and (**d**) 40%) at strain rate levels of 70/s, 100/s, and 140/s.

**Figure 15:**
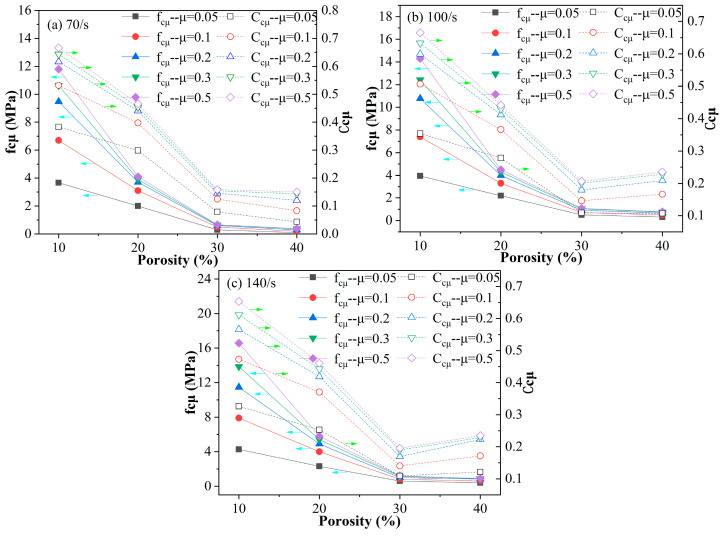
Variations in *f_cμ_* (the label blue arrows point to) and *C_cμ_* (the label green arrows point to) with porosity of the cellular concrete specimen at different strain rate levels ((**a**) 70/s, (**b**) 100/s, and (**c**) 140/s) when interface friction coefficient *μ* is set as 0.05, 0.1, 0.2, 0.3 and 0.5 respectively.

Based on the simulation of Section 3.2 (considering only the inertia effect), the interface friction coefficients *μ* between the specimen and pressure bar are set as 0.05, 0.1, 0.2, 0.3, and 0.5 in order to study the effects of interface friction on dynamic strength enhancement. The additional compressive strength increment *f_cμ_* of the reconstituted stress–strain curve in contrast to the counterpart in Section 3.2 is the dynamic strength enhancement induced only by the effect of interface friction, as shown in Figure 14 and Figure 15. The proportionality coefficient *C_cμ_* is formulated in Equation (2), which indicates the proportion of compressive strength enhancement induced by the interface friction effect to that of enhancement due to the whole structural effect (lateral inertia confinement effect and interface friction effect).

(2) $$C_{c\mu }=\frac{f_{c\mu }}{f_{c\mu }+f_{cl}}$$

As can be seen from Figure 14, the compressive strength increment *f_cμ_* induced only by the effect of interface friction increases with the increase in the interface friction coefficient *μ*. And the increasing trend is more obvious when *μ* is less than 0.3, while the *f_cμ_*-*μ* curves (especially for specimens with relatively high porosities of 30% and 40%) gradually become smooth when *μ* exceeds 0.3. The variational trend of *C_cμ_*-*μ* curves is in accord with that of *f_cμ_*-*μ* curves, as shown in Figure 14. It can be speculated that the proportion of the interface friction effect to the whole structural effect will not grow significantly with interface friction coefficient *μ* when it exceeds 0.3 for a specimen with a specific porosity at a given strain rate. As for the cellular concrete with a porosity of 10% or 20%, the coefficient *C_cμ_* values are close to each other at each given interface friction coefficient *μ* and strain rate level. However, for the cellular concrete with a higher porosity of 30% or 40%, there is a sudden increase in *C_cμ_* when the strain rate level rises from 70/s to 100/s. It manifests that the cellular concrete specimens with high porosities are less sensitive to the interface friction effect at a relatively low strain rate level. This may be ascribed to the attenuation effect of the inner pore structure on the interface friction effect, and there may exist a threshold strain rate for specimens with high porosities, above which the attenuation effect declines significantly.

The attenuation effect of the inner pore structure on the interface friction effect can be observed more obviously in Figure 15, in which *f_cμ_* and *C_cμ_* both decline rapidly with an increase in porosity at a given strain rate, and the curve obviously becomes smooth when porosity surpasses 30%. The value of *f_cμ_* drops to near zero, while the value of *C_cμ_* reaches a relatively low level of about 0.05~0.25 for a specimen with a porosity of 40%. The absolute increment in compressive strength *f_cμ_* caused by interface friction *μ* is almost negligible for a specimen with a porosity of 40% compared with a specimen with a porosity of 10%. Therefore, it is necessary to apply petroleum jelly and other means to reduce the friction coefficient of the contact face in the SHPB test for cellular concrete, especially for specimens with a relatively low porosity below 20%.

## 4. Conclusions

As a new kind of lightweight concrete, cellular concrete with millimeter-size isolated spherical pores can greatly reduce the transportation cost of concrete coarse aggregate in the construction of protection projects on remote islands. The previous research reveals that the compressive strength of this novel cellular concrete has an obvious strain rate effect, especially for specimens with high porosities such as 40%. This paper established numerical SHPB tests to investigate structural effects during the tests, attempting to reveal the mechanism of the strain rate effect of this kind of lightweight concrete. The main conclusions can be drawn as follows:

(1) The increment in compressive strength caused by lateral inertia confinement decreases from 5.9 MPa for a specimen with a porosity of 10% to 2 MPa for a specimen with a porosity of 40% at a strain rate level of 70/s, while the same decreasing trend is found at the other strain rate levels of 100/s and 140/s.
(2) The lateral inertia confinement effect inside the cellular concrete specimen can be divided into the elastic development stage and plastic development stage, bounded by the moment dynamic stress equilibrium is achieved.
(3) The increase in compressive strength caused by the lateral inertia confinement effect is mainly attributable to the propagation of the lateral confinement to the interior of the specimen, which starts from the plastic expansion of the plastic zone at the edge of the specimen.
(4) The proportion of the interface friction effect to the whole structural effect will not grow significantly with interface friction coefficient *μ* when it exceeds 0.3 for a specimen with a specific porosity at a given strain rate.
(5) The influence of the interface friction effect on the compressive strength of cellular concrete is attenuated due to the inner pore structure.

The results of this paper can help to obtain a better understanding of the enhancement mechanism of the compressive strength of cellular concrete. Moreover, they can guide us to obtain the genuine strain rate effect of compressive strength for cellular concrete, with which a more reasonable result can be obtained in structure design. Thus, further research work on this novel cellular concrete may be focused on the simulation method in structure design.

## Figures and Tables

**Table 1:** Parameters of the finite element model for the cellular concrete specimens.

Pore Diameter mm	Theoretical Porosity %	Actual Porosity %	Simulated Porosity %	Cell Size mm	Pore Number	Unit Number
5	10	9.5	9.5	8.7656 × 8.7656 × 8.7656	196	84,672
	20	19.3	19.7	7.000 × 6.8930 × 6.8930	405	88,290
	30	27.8	29.2	5.9033 × 6.1667 × 6.1667	600	76,800
	40	38.7	38.5	5.700 × 5.4624 × 5.4624	792	101,376

## Data Availability

The original contributions presented in the study are included in the article, further inquiries can be directed to the corresponding author.
